# Ovotransferrin Fibril—Gum Arabic Complexes as Stabilizers for Oleogel-in-Water Pickering Emulsions: Formation Mechanism, Physicochemical Properties, and Curcumin Delivery

**DOI:** 10.3390/foods13091323

**Published:** 2024-04-26

**Authors:** Zihao Wei, Yue Dong, Jingyu Si

**Affiliations:** State Key Laboratory of Marine Food Processing & Safety Control, College of Food Science and Engineering, Ocean University of China, Qingdao 266404, China

**Keywords:** ovotransferrin fibrils—gum arabic complexes, oleogel-in-water Pickering emulsion, formation mechanism, physicochemical properties, curcumin delivery

## Abstract

This project aimed to explore the influence of the interaction between ovotransferrin fibrils (OTF) and gum arabic (GA) on the formation mechanism, physicochemical properties, and curcumin delivery of the oleogel-in-water Pickering emulsion. Cryo-scanning electron microscopy results showed that OTF—GA complexes effectively adsorbed on the oil–water interface, generating spatial hindrance to inhibit droplet coalescence. The texture analysis also proved that OTF—GA complexes endowed oleogel-in-water Pickering emulsion with preferable springiness (0.49 ± 0.03 mm), chewiness (0.43 ± 0.07 mJ), and adhesion (0.31 ± 0.01 mJ). By exploring the coalescence stability, droplet size, and rheological properties of OTF—GA complexes–stabilized oleogel-in-water Pickering emulsion (OGPE), the higher coagulation stability, larger average droplet size (46.22 ± 0.08 μm), and stronger gel strength were observed. The microrheological results also exhibited stronger attraction between the OGPE droplets, a more pronounced solid-like structure, and a slower speed of movement than OTF-stabilized oleogel-in-water Pickering emulsion (OPE). Meanwhile, OGPE significantly enhanced the extent of lipolysis, stability, and bioaccessibility of curcumin, suggesting that it possessed superior performance as a delivery system for bioactive substances. This project provided adequate theoretical references for protein–polysaccharide complexes–stabilized oleogel-in-water Pickering emulsion, and contributed to expanding the application of oleogel-in-water Pickering emulsion in the food industry.

## 1. Introduction

Protein fibrils are one of the most typical protein structures that can be obtained from protein by prolonged heat treatment in acidic conditions, playing crucial roles in achieving developed physiochemical and biological properties [1,2]. Most studies on protein fibrils focus on non-transferrin, such as β-lactoglobulin fibrils, α-lactalbumin fibrils, and rice glutelin fibrils; however, the lack of iron ions restricts its application in functional foods [3]. In addition, the longer protein fibrils lengths, which are typically micron-sized, create obstacles in the design of food delivery systems [4].

Ovotransferrin fibrils (OTF) are iron-binding protein fibrils derived from egg white protein, which exhibit excellent anti-inflammatory, antibacterial, and oxygen ion scavenging activity by binding with iron ions [5]. Our preliminary experiments have demonstrated that OTF have no in vitro cytotoxicity, laying the foundation for their large-scale application in the food industry [6]. Additionally, OTF are relatively short and flexible, which facilitates the accurate design of food delivery systems [4,7]. Among various transportation vehicles, Pickering emulsions exhibit various advantages, such as outstanding dispersibility, environmental friendliness, targeted delivery and controlled release; thus, Pickering emulsions are widely used in the fields of food, pharmaceuticals, and cosmetics. Oleogel-in-water Pickering emulsion delivery systems combine the advantages of both oleogels and emulsions, improving the water dispersibility of the oleogel system while reducing the content of saturated fatty acids and trans fatty acids, thereby enhancing the organoleptic quality of the food substrates [8]. During Pickering emulsion formation, the stabilizer particles adsorb at the interface of the two phases and form a mechanical barrier, triggering a change in the inter-particle spatial site resistance and helping to regulate the physicochemical properties of the Pickering emulsions [9].

In recent years, relevant studies have demonstrated that the addition of polysaccharides could alter the molecular structure of proteins, endowing fibrils with a more complex morphology and superior emulsification characteristics. Meanwhile, the establishment of the protein fibril–polysaccharide complexes also provides a bridge to load bioactive compounds, allowing them to be maintained in complicated food matrices [10,11]. Among numerous polysaccharides, gum arabic (GA) is widely used in food industries as an adhesive, emulsifier, and thickener due to its superior water dispersibility and low viscosity [12]. GA can form complexes with proteins through electrostatic interactions, in which hydrophobic peptide chains are adsorbed at the oil–water interface, while hydrophilic carbohydrate fragments extend to the continuous phase, forming an uninterrupted interface layer [13]. This multi-layer structure of protein–polysaccharide helps to improve the stability of emulsion systems under harsh conditions, thereby achieving the efficient delivery of bioactive compounds [14].

In our previous studies, the interaction of GA with OTF and the effect of the addition of GA on the morphology and properties of OTF have been elucidated. Driven by electrostatic interactions, we found that GA became stacked as spherical clusters, wrapped around the OTF to form a specific knot-like structure [15]. Although the microstructure and physicochemical properties of OTF—GA complexes have been studied, a lack of its application in food-grade delivery systems remains. Curcumin is a diketone compound extracted from turmeric, which is extensively used in the field of functional foods and biomedicine due to its hypolipidemic, antitumor, anti-inflammatory, choleretic, and antioxidant effects. Nevertheless, it is difficult to retain curcumin activity for further absorption owing to its poor stability and low bioaccessibility [16,17]. Therefore, it is meaningful to choose suitable carriers for the encapsulation and protection of curcumin, which contribute to maximizing its bioaccessibility [18].

In summary, this study aimed to explore the influence of OTF—GA complexes on the formation mechanism, physicochemical properties, and curcumin delivery of oleogel-in-water Pickering emulsion. First, the formation mechanisms of OTF—GA complexes–stabilized oleogel-in-water Pickering emulsion (OGPE) and OTF-stabilized oleogel-in-water Pickering emulsion (OPE) were revealed, based on the microstructural images. Subsequently, the physicochemical properties of OGPE and OPE were determined by analyzing their droplet sizes, textural properties, and microrheological behavior. Finally, OPE and OGPE served as the delivery system to investigate the protective and release effects of curcumin.

## 2. Materials and Methods

### 2.1. Materials

The emulsifiers in Pickering emulsion were composed of ovotransferrin (an assay of no less than 88%) and gum arabic, which were procured from Neova Technologies Inc. (Abbotsford, BC, Canada) and Macklin Co., Ltd. (Shanghai, China). The oil phase in Pickering emulsion consisted of canola oil and candelilla wax, which were supplied by Yihai Kerry Food Marketing Co., Ltd. (Shanghai, China) and Aladdin Co., Ltd. (Shanghai, China). Curcumin (purity over 95%) was acquired from Ryon Biotechnology Co., Ltd. (Shanghai, China). Pepsin (3 × 10^3^ U/g) and pancreatin (4 × 10^3^ U/g) were purchased from Solarbio Biotechnology Co., Ltd. (Beijing, China). Fluorescent dyes (Nile red and Nile blue A) were procured from Yuanye Biotechnology Co., Ltd. (Shanghai, China). Pig bile salt, calcium chloride, and sodium chloride were of analytical grade and were obtained from Sinopharm Chemical Reagents (Shanghai, China).

### 2.2. Preparation of OTF—GA Complexes

OTF were first prepared according to the procedure described by Wei et al. [15]. Ovotransferrin solution was obtained by fully dissolving ovotransferrin into the pH 2 of water containing 100 mM of sodium chloride. Afterwards, the pH of the ovotransferrin solution was regulated to 2 and incubated at 90 °C for 24 h under magnetic stirring to obtain the protein fibrosis to prepare the OTF coarse suspension. Then, the OTF coarse suspension was removed to an ice bath to stop the fibrosis. After the pH of the OTF coarse suspension was again adjusted to 2, the OTF suspension was finally obtained by dialysis for 24 h. OTF—GA complexes with a total biopolymer concentration of 50 mg/mL were prepared by blending the GA solution and the OTF suspension in equal proportions under magnetic stirring (600 rpm, 6 h) and adjusting the pH of the mixed solution to 4.6.

### 2.3. Construction of OPE and OGPE

Ovotransferrin fibrils—gum arabic (OTF—GA) complexes–stabilized oleogel-in-water Pickering emulsion (OGPE) was prepared by following the methods of an existing reference, with slight modifications [19]. Specifically, candelilla wax was mixed with canola oil at different mass fractions (from 1.0–1.5%, with a gradient of 0.1%) under continuous stirring at a high temperature for 5 min until the candelilla wax completely disappeared. After the mixed solution was cooled to ambient temperature, it was stored at 4 °C to accelerate oleogel formation. In addition, the sample was inverted at ambient temperature for 0.5 h, with a lack of movement as a sign of the successful preparation of oleogel. Oleogel (65% volume fraction) was combined with OTF—GA complex suspension (50 mg/mL, pH 4.6), and the mixture was sheared at 10,000 rpm for 3 min using a T25 high-speed shear dispersing machine (IKA-Werke GMBH & CO., Staufen, Germany) to acquire OGPE. OTF-stabilized oleogel-in-water Pickering emulsion (OPE) was regarded as a control to characterize the emulsions.

### 2.4. Droplet Size of OPE and OGPE

The droplet sizes of the emulsions were determined using a Bettersize2600 laser particle size distributor (Dandong Bettersize Instruments Co., Ltd., Dandong, China). The OPE and OGPE samples were slowly injected into the metal sample reservoir, and the shading rate was controlled to stabilize within 10–15%. The results were presented by the volume mean diameter (D_4,3_) [14].

### 2.5. Coalescence Stability of OPE and OGPE

The coalescence stability of the emulsion was indicated by the dynamic change between the droplet size of the system in 1% anionic surfactant and a function of time. The droplet size of the system in 1% anionic surfactant was measured using a Bettersize2600 laser particle size distributor, before and after storage of 20 days.

### 2.6. Confocal Laser Scanning Microscope (CLSM)

The microstructure of OPE and OGPE was observed by CLSM (A1R HD25, ECLIPSE Ni-E, Nikon Corporation, Tokyo, Japan). First, Nile red dye solution and Nile blue A dye solution were added to the emulsion and thoroughly mixed. Then, an appropriate amount of stained emulsion was placed on the slide and slowly covered with the cover slide to avoid bubbles. Fluorescence signals of the oil phase and OTF—GA complexes were excited at 488 nm and 633 nm, respectively. The image resolution is 1270 × 408, the width if 1270 pixels, the height is 408 pixels, and the image scale is 20 μm [20].

### 2.7. Cryo-Scanning Electron Microscope (CryoSEM)

The CryoSEM (helios5, ThermoFisher, Waltham, MA, USA) was used to confirm the structural state of the oil–water interface of OPE and OGPE. The emulsion sample was placed on the sample stage and quickly frozen in liquid nitrogen, then moved to the sample processing bin and frozen broken. Afterwards, the sample was sublimed at −90 °C for 10 min, and gold was sprayed onto the sample for 30 s under 10 mA of current. The accelerating voltage was 1 kV, and the working distance was 10 mm. Finally, the microscopic images of the emulsion were acquired under different magnifications, including ×2000 and ×50,000 [21].

### 2.8. Rheological and Microrheological Behavior

A Physica MCR 301 with a Peltier plate temperature controller (Anton Paar, Graz, Austria) was used to determine the rheological properties of the emulsion samples. All tests were performed on a 50 mm parallel plate (PP50) with a gap height of 0.75 mm, and the temperature was set at 25 °C [22]. First, the strain sweep was drawn to characterize the relationship between the strain level, the storage modulus (G′), and the loss modulus (G″). Then, a frequency sweep was used to test the frequency correlation curve of G′ and G″ within the frequency scope of 0.5–25 Hz at a 0.1% strain level. Additionally, the viscosity of the system was examined by increasing the shear rate from 0.01 to 200 s^−1^.

The microrheological properties of OPE and OGPE were investigated using a Rheolaser Master device (Formulaction, Toulouse, France). The emulsion sample was added to the sample pool and tested for 2 h at 25 °C to obtain the relevant parameters, including the mean square displacement (MSD), the elasticity index (EI), the macroscopic viscosity index (MVI), the solid–liquid balance (SLB), and the fluidity index (FI) [23].

### 2.9. Texture Test

The hardness, elasticity, cohesiveness, gelatinousness, and adhesiveness, as well as the chewiness of the samples, were evaluated using a texture tester (Food Technology Corporation, Sterling, VA, USA) by using a cylindrical probe with a 30 mm diameter. The test conditions were as follows: the initial force and deformation percentage of the sample were chosen to be 0.2 N and 70%. The height of the probe raised above the sample surface was set at 20 mm, and the test speed was selected at 30 mm/min.

### 2.10. Establishment of Curcumin-Loaded OPE and OGPE

The preparation method of curcumin-loaded OPE and OGPE was developed from our previous work [24]. Firstly, canola oil containing curcumin (1 mg/mL) was stirred magnetically and heated by stirring it through an oil bath to promote the dissolution of curcumin in the canola oil, thereby obtaining curcumin-loaded canola oil. Subsequently, candelilla wax was added to the curcumin-loaded canola oil at a critical gel concentration and stirred with heat for 5 min to promote the complete dissolution of the candelilla wax in the curcumin-loaded canola oil. The mixed solution was held at ambient temperature and gradually cooled down until the curcumin-loaded oleogel was obtained. Eventually, the OTF suspension and the OTF—GA complexes suspension (50 mg/mL, pH 4.6) were separately mixed with the curcumin-loaded oleogel. Next, a T25 high-speed shear dispersing machine was used to shear the above mixtures at 10,000 rpm for 3 min to prepare curcumin-loaded OPE and OGPE.

### 2.11. UV Protection of Curcumin by OPE and OGPE

The UV protection properties of curcumin obtained from OPE and OGPE were recorded at different time points (0–6 h, interval of 1 h) on the UV radiation equipment [7]. Specifically, 100 μL of the OPE and OGPE samples, with a fixed processing time, were mixed with 1.2 mL of ethanol, followed by centrifugation at 12,000 rpm for 20 min. After centrifugation, the supernatant was removed, and ethanol was again added for curcumin extraction; the above steps were repeated until the clear and transparent supernatant was obtained. After merging the supernatant and diluting the sample to an appropriate gradient, the absorption spectra of OPE and OGPE were recorded using a UV–Vis spectrophotometer at 426 nm; then the residual curcumin level was acquired according to the equation below:

(1) $$\mathrm{Residual}\;\mathrm{curcumin}\;\mathrm{level}\%=C/C_{0}\times 100$$

where *C* indicated the content of curcumin at the different processing times, and *C*_0_ expressed the initial content of curcumin.

### 2.12. In Vitro Simulated Gastrointestinal Digestion Experiments

The simulated gastrointestinal digestion process was derived from a previously constructed method, with some modifications [25]. Simulated gastric juice (SGJ) was obtained by dissolving NaCl in deionized water (2:1, *v*/*v*) and regulating the pH to 1.2. A total of 16 mL of SGJ was added to the OPE and OGPE samples containing 2 g of oil and mixed uniformly in a 37 °C oil bath. Subsequently, 4 mL of SGJ containing pepsin was added to the above mixture to initiate 2 h of gastric digestion. Gastric digestion was stopped by changing the pH of the mixture to 7.2.

In the next experiment regarding intestinal digestion, simulated intestinal fluid (SIF) was first obtained by mixing Tris, calcium chloride, and pig bile salts in water (20 mL) and regulating the pH to 7.5. SIF containing pancreatin was mixed with the digesta after gastric digestion to conduct 2 h of intestinal digestion in a 37 °C oil bath. During SIF, the pH of the mixture would decrease owing to the generation of free fatty acids (FFAs), which could be maintained at 7.5 by adding 0.25 mol/L of NaOH. The rate of FFAs released was calculated according to the equation shown below:

(2) $$\mathrm{FFAs}\%=\frac{C_{\mathrm{NaOH}}{\;\times \;V}_{\mathrm{NaOH}}{\;\times \;M}_{\mathrm{lipid}}}{w_{\mathrm{lipid}}\;\times \;2}\times 100$$

where *C*_NaOH_ indicated the molar concentration of NaOH, *V*_NaOH_ represented the volume of NaOH, *M*_lipid_ indicated the molecular weight of canola oil, and *w*_lipid_ represented the mass of the original weight of the canola oil.

### 2.13. Bioaccessibility of Curcumin

After digestion treatment, the digesta was centrifuged at 11,000 rpm for 45 min. The intermediate micellar phase produced after centrifugation was removed and mixed with anhydrous ethanol at a 1:1 volume ratio. The supernatant resulting from the centrifugation (10,000 rpm, 20 min) of the above mixture was removed. The absorbance value of the supernatant diluted with anhydrous ethanol was measured at 426 nm. After the absorbance value was substituted into the standard curve, the bioaccessibility of curcumin was acquired using the following formula:

(3) $$\;\mathrm{Bioaccessibility}\;\%=\frac{\mathrm{the}\;\mathrm{content}\;\mathrm{of}\;\mathrm{curcumin}\;\mathrm{in}\;\mathrm{micelles}}{\mathrm{The}\;\mathrm{total}\;\mathrm{amount}\;\mathrm{of}\;\mathrm{curcumin}\;\mathrm{in}\;\mathrm{the}\;\mathrm{emulsions}}\;\times \;100\;$$

### 2.14. Statistical Analysis

All data were displayed as mean ± standard deviation (*n* ≥ 3). Origin 2023 software (Northampton, MA, USA) was employed for graph construction. SPSS 22.0 software was used for statistical analysis (*t*-test, *p* < 0.05 indicates significant difference).

## 3. Results and Discussion

### 3.1. Droplet Size and Coalescence Stability of OPE and OGPE

For the stability of the emulsions, the addition of emulsifier must be able to reduce the interfacial tension between the oil and water phases and prevent the recoalescence of the oil droplets [26]. Natural emulsifiers, such as protein–polysaccharide complexes, have been proven to heighten the stability of Pickering emulsions, which may be related to several parameters. One parameter may be the mechanical properties of the emulsions, where sufficient complexes cover the oil–water interface and form a solid mechanical barrier on the interface, which can provide good spatial protection for emulsion droplets and enhance the spatial stability of emulsion [27,28]. Another parameter may be the rheological properties of emulsions. The presence of complexes can enhance the viscosity of the aqueous phase, further postponing the movement of droplets and heightening the solid-like properties of the Pickering emulsion system [29].

As shown in Figure S1 (see Supplementary Material), the average droplet size of OGPE (46.22 ± 0.08 μm) was larger than that of OPE (44.45 ± 0.14 μm), indicating that the interaction between OTF and GA affected the average drop sizes of the emulsion. The binding sites of protein were heavily exposed by chemical treatment and attracted polysaccharides to combine with them, which was beneficial for increasing the droplet size of the emulsions [30]. However, OGPE exhibited a narrower distribution of droplet sizes and a higher count intensity than did OPE. This might be due to the excellent interfacial coverage of the OTF—GA complexes with sufficient concentration, which acted as stabilizers of OGPE and could be adequately mixed with the oil phase during the process of emulsification to more effectively envelop the oil phase, thus forming more droplets [31,32].

**Figure 1:**
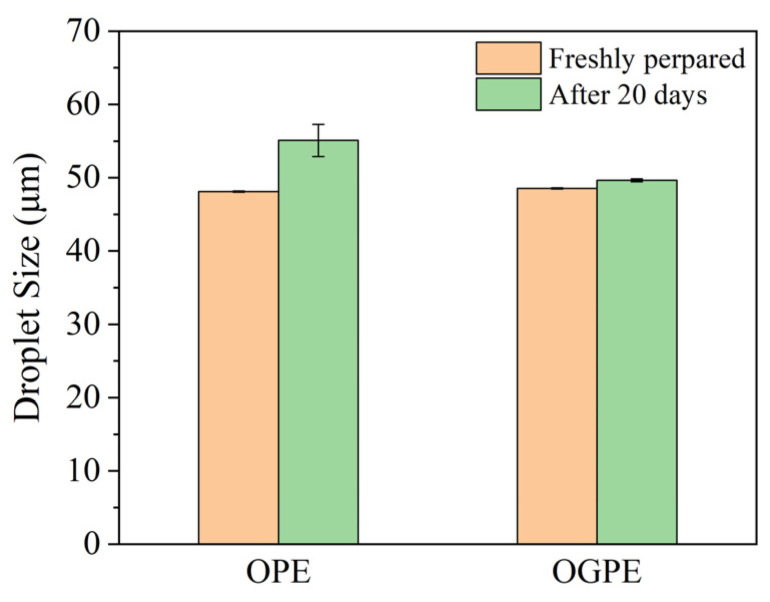
Droplet size of OPE and OGPE.

The change in the droplet size of the emulsion in the anionic surfactant with storage time reflected the condensation stability of the emulsion. Meanwhile, the presence of anionic surfactant may inhibit the flocculation of the emulsion droplets, thereby maintaining the separation of individual droplets in the emulsions [33]. The droplet sizes (D_4,3_) of OPE and OGPE, before and after 20 days of storage, are shown in Figure 1. The size of the droplets of OGPE showed few changes during the 20-day storage compared to the size of the OPE droplets, indicating that OGPE exhibited high coacervation stability. Since the coacervation stability of the emulsion droplets was closely related to the viscoelasticity of the interfacial film, OTF—GA complexes might form a more viscoelastic interfacial film, which was helpful for the inhibition of the condensation of emulsion droplets. In addition, sufficient OTF—GA complexes could be adsorbed on the oil–water interface, forming a denser protective barrier through effective accumulation, playing a steric hindrance role and reducing the condensation of the emulsion droplets [28].

### 3.2. Forming Mechanism of OPE and OGPE

**Figure 2:**
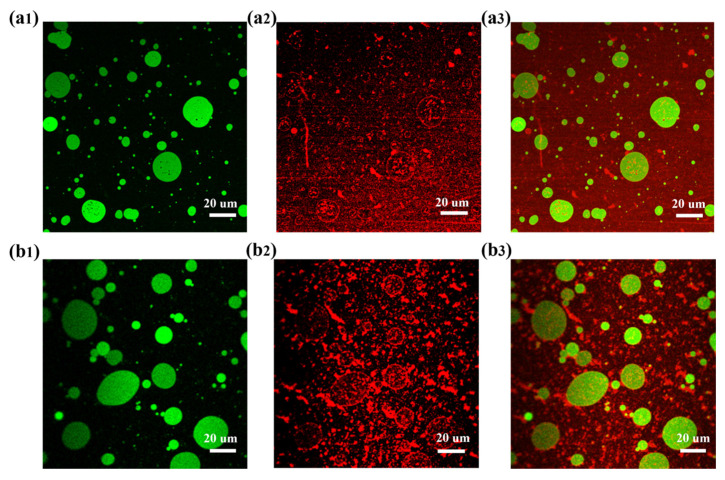
CLSM images of OPE (**a1**–**a3**) and OGPE (**b1**–**b3**). (**a1**,**b1**) The oil phase stained with Nile red is shown in green. (**a2**,**b2**) The OTF-GA complexes dyed with Nile blue A are shown in red. (**a3**,**b3**) The overlap channel.

To elaborate the forming mechanism of OPE and OGPE, the interfacial characteristics of the oleogel-in-water Pickering emulsions were examined using CLSM and CryoSEM. The interface structure information for the Pickering emulsion can be obtained more directly by CLSM, thereby verifying the effective adsorption of stabilizers at the oil–water interface. The green circular area corresponded to the oil phase after Nile red staining, and the red fluorescent luminescent layer represented stabilizers after Nile blue A staining. As could be seen from Figure 2, the appearance of the green circular area representing the oil phase indicated that the oil phase was located inside the droplet, which suggested that OPE and OGPE were indeed O/W type Pickering emulsions. Furthermore, it was observed that a large number of fibrils matrices surrounded the emulsion droplets, indicating that OTF and OTF—GA complexes could effectively adsorb on the surface of the emulsion droplets to stabilize OPE and OGPE [4].

**Figure 3:**
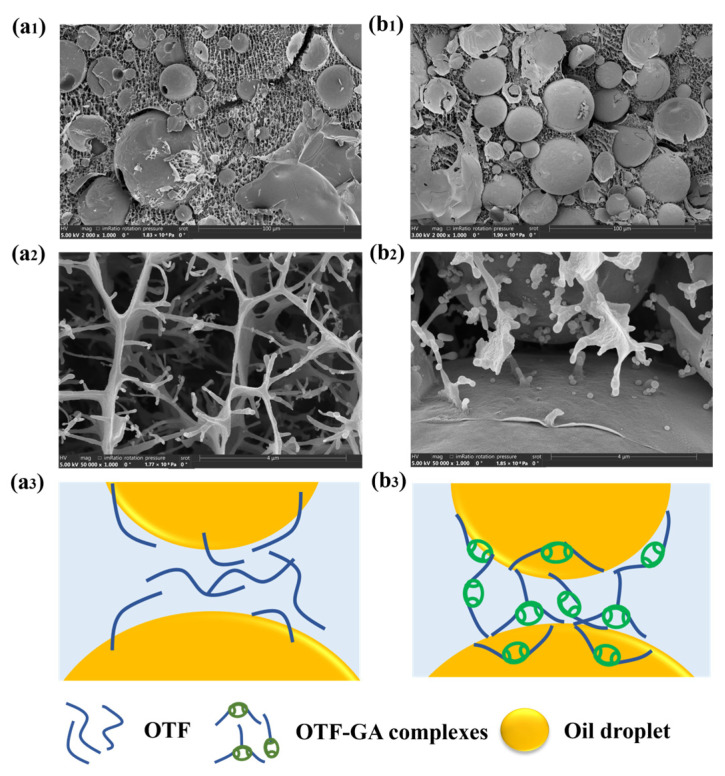
CryoSEM images of OPE (**a**) and OGPE (**b**) at different magnifications, including ×2000 (**a1**,**b1**) and ×50,000 (**a2**,**b2**). The stabilization mechanism of OPE (**a3**) and OGPE (**b3**).

The micromorphology of the emulsions was further observed by CryoSEM, which was helpful for exploring the stabilization mechanism of the emulsions. Due to the removal of water from the sample during the sublimation process prior to CryoSEM observation, only solid particles and oil phases were present in the image. Figure 3(a1,a2) shows CryoSEM images of OTF-stabilized OPE, and Figure 3(b1), as well as Figure 3(b2), shows CryoSEM images of OTF—GA complexes-stabilized OGPE. As can be seen from Figure 3, there was network structure between the droplets of both emulsions, which was due to the presence of OTF in both emulsions. At the interface of OGPE, the OTF—GA complexes formed by the combination of OTF and GA, with a spherical particle structure, was adsorbed on the droplet surface for stabilizing the emulsion [34]. The stabilization mechanism of the two emulsions is shown in Figure 3(a3,b3). Firstly, the independent oil and water phases were quickly blended under the action of external mechanical forces, forming smaller oil droplets. Then, the protein fibrils were anchored and adsorbed on the surface of the droplets, forming a hard mechanical barrier on the oil–water interface. Finally, the fibrils matrix adsorbed on the surface of the droplets or aggregated in continuous phases bridge with each other to build a stable network structure [35].

### 3.3. Rheological Analysis and Texture Properties

**Figure 4:**
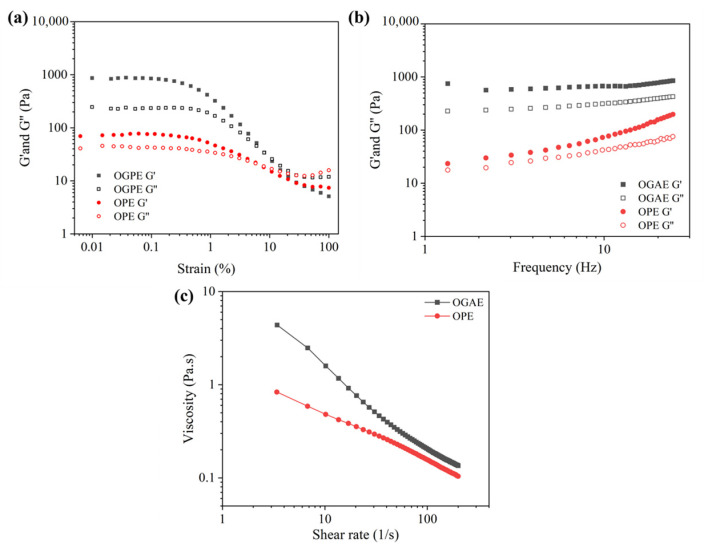
Strain sweep (**a**), frequency sweep (**b**), and viscosity (**c**) of OPE and OGPE.

Figure 4 showed the rheological properties of OPE and OGPE. First, a strain sweep was performed on OPE and OGPE to determine the linear viscoelastic region. Figure 4a shows the strain sweep curves of OPE and OGPE. In the linear viscoelastic region, the energy storage modulus (G′) and the loss modulus (G″) of the two emulsions were independent of the applied strain. At low strain values, the G′ of the two emulsions was greater than G″, showing elastic behavior. With the increase in strain value, both G′ and G″ decreased, and G″ displayed an upward trend after decreasing. When the strain value increased to a certain value, G″ was greater than G′, indicating that the interfacial film structure of the emulsion droplet was deformed under high stress value, and the initial elastic behavior was transformed into viscous behavior [36]. The strain values in the linear viscoelastic region were selected for frequency sweep. As can be seen from Figure 4b, the G′ of OPE and OGPE was greater than the G″, indicating that both emulsions had an elastic gel structure. The higher G′ of OGPE compared to OPE meant that OGPE exhibited stronger elastic gel strength [37].

As shown in Figure 4c, OGPE achieved a higher viscosity than did OPE. The reason was that the OTF—GA complex suspension with polysaccharide added exhibited higher viscosity compared with that of the OTF suspension, which could be used as the thickener of the system. The excess OTF—GA complexes that were not adsorbed on the oil–water interface remained in the continuous phase, which had a thickening effect on the prepared emulsion and enhanced the anti-deformation ability of the droplets, thereby leading to higher G′ and viscosity [4]. In addition, as shown in Figure 4c, the viscosity of OPE and OGPE gradually decreased as the shear rate increased from 0.01 to 200 s^−1^, showing shear thinning. This was mainly because the emulsion droplets were orderly distributed along the flow direction as the shear rate increased, thereby reducing the flow resistance and viscosity [38].

**Figure 5:**
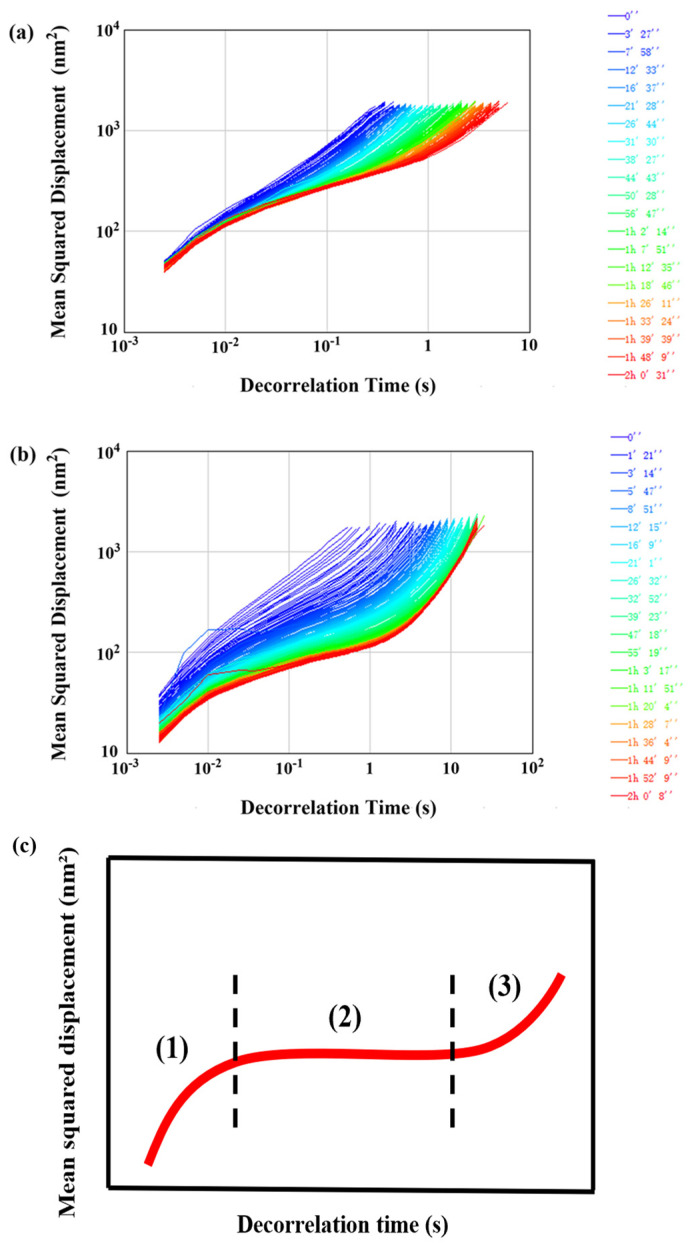
Mean squared displacement of OPE (**a**), OGPE (**b**), and viscoelastic emulsions (**c**).

Compared with traditional rheology, microrheology can monitor the viscoelastic and other microrheological properties of samples, without interference or destructiveness [38]. The changes of mean square displacement (MSD) of the two emulsions with decorrelation time were obtained by using a Rheolaser Master optical microrheometer. The particle MSD curve increased linearly with time in a completely viscous fluid. In contrast, the MSD of the particles in a viscoelastic fluid was limited, and the particles were stuck in a network structure, establishing a plateau region [39]. Based on the particle motion trajectories at different decorrelation times, they can be divided into three main behaviors (Figure 5c): (1) At the beginning of the decorrelation time, the particles, which were only affected by solvent viscosity, moved freely inside the cage. The increase in MSD was linearly related to the decorrelation time. (2) At the middle of the decorrelation time, the particle motion was affected by the interactions of the cage structure, resulting in a slower the movement and reaching the plateau region. (3) At the end of the decorrelation time, the particle motion was no longer affected by the cage, and the MSD slope increased linearly with the decorrelation time curve [40]. As shown in Figure 5a,b, both the OTF-stabilized OPE and the OTF—GA complexes–stabilized OGPE clearly showed a plateau in the MSD curves, which may be due to the limited movement of the droplets in the emulsions, forming a denser viscoelastic structure [40]. Compared with OPE, OGPE exhibited a larger de-correlation time scale, indicating that OGPE exhibited higher viscoelasticity.

**Figure 6:**
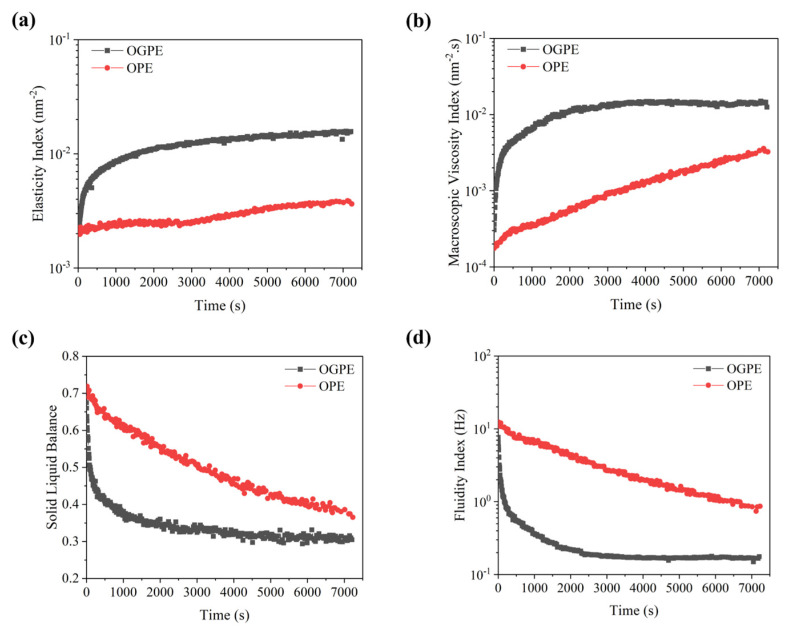
Elasticity index (**a**), macroscopic viscosity index (**b**), solid–liquid balance (**c**), and fluidity index (**d**) of OPE and OGPE.

The values of the elasticity index (EI), the solid–liquid balance (SLB), the macroscopic viscosity index (MVI), and the fluidity index (FI) were further calculated from the MSD curves using Rheosoft Master 1.4.0. The slope value of the elastic plateau region of the MSD curve was called the solid–liquid balance (SLB). It reflected the equilibrium state of the sample, indicating the liquid or solid sample characteristics. The elasticity index (EI) was the reciprocal of the height of the plateau region, which could be used to characterize the elastic nature of the sample [41]. The MVI symbolized the slope of the MSD curve behind the platform area, which was the viscosity of the emulsion in the zero shear state. The sample with a higher macroscopic viscosity value (MVI) value indicated that the emulsion droplet took more time to move a certain distance. The fluidity index (FI) represented the reciprocal of the decorrelation time, which was used to represent the fluidity of the emulsion droplets [41]. Figure 6 shows the EI, MVI, SLB, and FI of the two emulsions. The EI of OGPE was greater than the OPE, which indicated that the OGPE showed a more obvious elasticity, as well as a strong attraction between the droplets (van der Waals force and hydrogen bond interaction). The SLB of both emulsions decreased over time, and OGPE had a lower SLB, which indicated that OGPE had a more obvious solid-like structure. In addition, the MVI of OGPE was larger than that of OPE, indicating that the viscosity of OGPE was larger. This was attributed to the higher viscosity of OGPE, which slowed down the movement speed of the emulsion droplets. The high viscosity also helped to inhibit the phase separation of the system, resulting in excellent stability [42].

The hardness, cohesiveness, springiness, gumminess, and adhesiveness, as well as the chewiness of OPE and OGPE were tested using a texture tester. As shown in Table 1, OGPE exhibited outstanding elasticity, chewability, and adhesiveness compared to OPE. The more prominent elasticity of OGPE was consistent with the results of the rheological analysis. The addition of GA promoted the chewability and adhesion of the emulsion. There were no significant differences in hardness, cohesiveness, and gumminess between the two emulsions. Based on the above analysis, OGPE exhibited a better application basis compared with OPE.

### 3.4. UV Protection of Curcumin by OPE and OGPE

**Figure 7:**
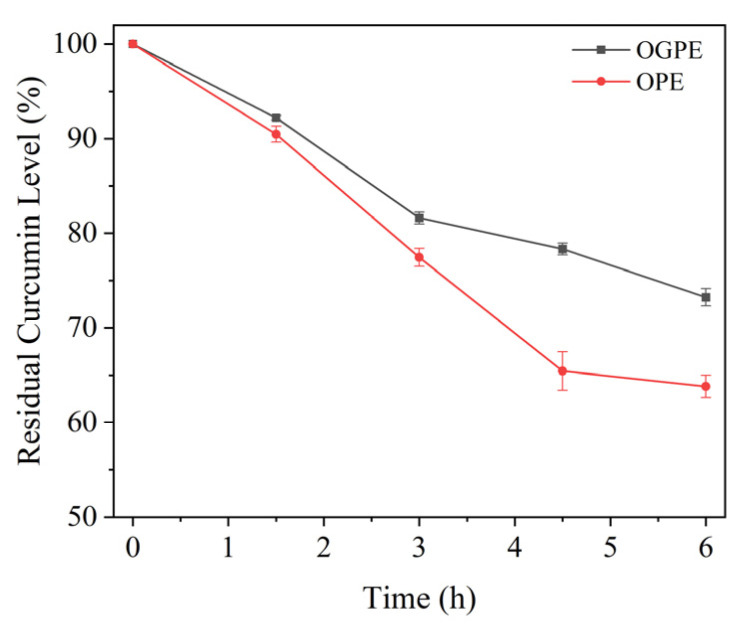
Residual curcumin levels in OPE and OGPE after UV radiation.

The delivery system represented by emulsion technology can provide excellent protection effects for curcumin under UV radiation. Figure 7 shows curcumin residue levels in OPE and OGPE after exposure to UV light. It can be seen from the figure that after UV treatment, the curcumin residue levels in both emulsions decreased, and the degradation rate of curcumin in OGPE was slower than that of the OPE, which suggested that OGPE could delay the dissolution of curcumin. This could be attributed to the following explanation. In OGPE, OTF—GA complexes composed of OTF and GA were wrapped around the oil droplet through complexation, which could improve the mechanical strength of the adsorption layer at the oil–water interface of the emulsion, as well as increase the thickness and density of the interface protective layer. This helped to provide a stronger physical barrier for curcumin in the oil phase and to delay the degradation of curcumin under UV light, thereby resulting in higher curcumin residue levels [35,43]. In addition, the higher viscosity of OGPE compared to that of OPE was also instrumental in protecting curcumin. The high viscosity led the emulsion droplets to accumulate closer together, limiting mobility and reducing the exposure of curcumin to UV light, resulting in higher curcumin residue levels in OGPE. This result was consistent with the previous report stating that molecular size, size distribution, and texture characteristics of polymer particles were important factors affecting the UV shielding ability of Pickering emulsions [44].

### 3.5. Lipolysis Degree and Bioaccessibility of Curcumin in OPE and OGPE

**Figure 8:**
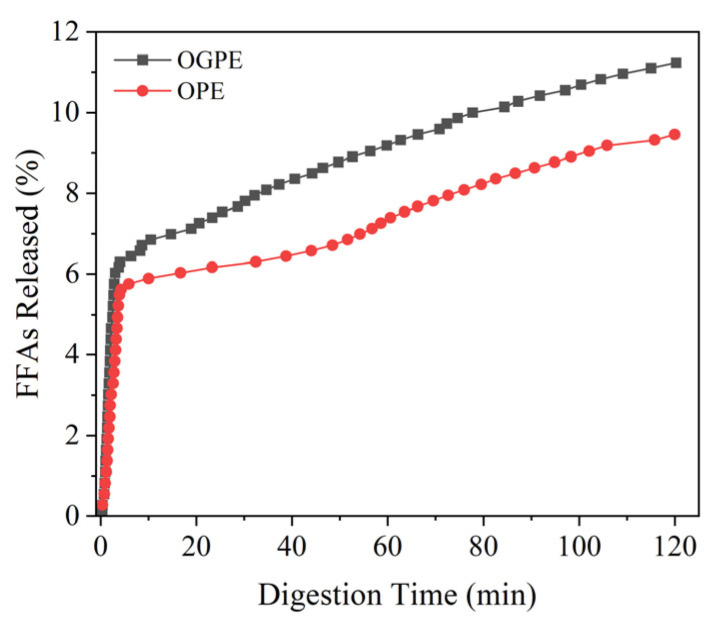
Release profile of free fatty acids (FFAs) in OPE and OGPE.

The lipolysis degree of the two emulsions is shown in Figure 8. After simulated intestinal digestion, OGPE released more free fatty acids (FFAs) than did OPE, indicating that OGPE stabilized by OTF—GA complexes had a higher lipolysis degree. The above phenomenon could be elucidated by the following reasons. Firstly, the stability of the OTF—GA complexes was affected by the surrounding environment. In the simulated gastric fluid, the low acid environment inhibited the carboxyl dissociation of GA and weakened the electrostatic interaction between GA and OTF, which resulted in the expansion of the OTF—GA complexes and exposed the wrapped oil phase, thus facilitating the contact between lipase and the oil phase. Secondly, the pH environment of simulated intestinal fluid was not conducive to the binding between GA and OTF, which further damaged the structure of the OTF—GA complexes and promoted the full contact between the oil phase and lipase to generate FFAs [45].

**Figure 9:**
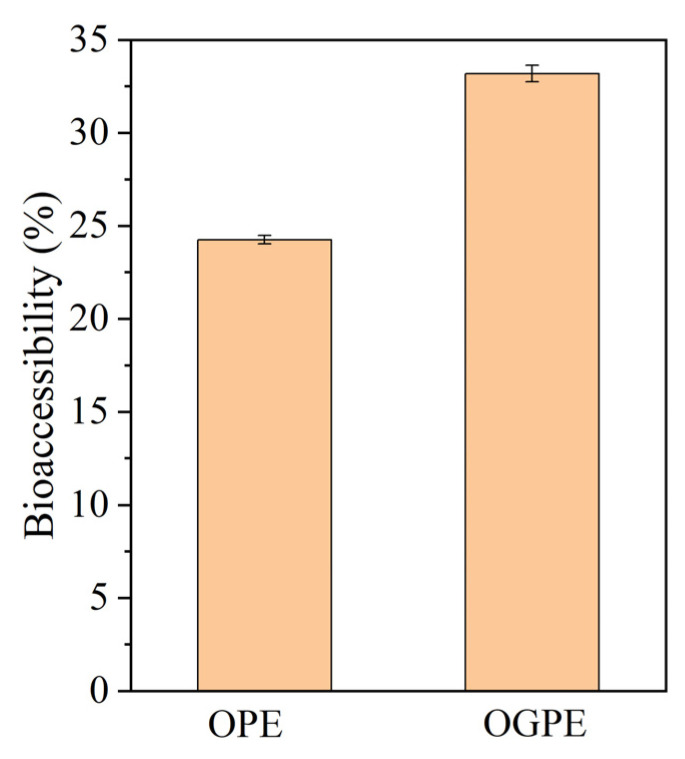
The bioaccessibility of curcumin in OPE and OGPE after in vitro digestion.

Figure 9 depicts the bioaccessibility of curcumin in OPE and OGPE after simulated digestion in vitro. As can be seen from the figure, the bioaccessibility of curcumin in OGPE was higher than that in OPE. Curcumin is not soluble in aqueous gastrointestinal fluids due to its poor water solubility. In order for curcumin to be absorbed through the GI tract, it must first be dissolved in the mixed micelle [46]. The generation of mixed micelles occurs due to the lipolysis of the emulsion. More FFAs were released by OGPE during lipolysis to generate more mixed micelles, which resulted in the better solubilization of curcumin, thereby improving the bioaccessibility of hydrophobic curcumin. In addition, the bioaccessibility of curcumin was closely related to the average droplet size of the emulsions. A smaller average droplet size corresponded to a larger specific surface area, which would result in more curcumin being exposed to a stringent external environment. Moreover, compared to OTF, OTF—GA complexes could form a hard interface layer to delay the degradation of curcumin and protect it from external oxidative stimulation [47].

## 4. Conclusions

In this paper, OGPE was innovatively constructed, and the effect of OTF—GA complexes on the formation mechanism, physicochemical properties, and curcumin delivery of oleogel-in-water Pickering emulsion was further investigated. The texture analysis revealed that the addition of GA significantly promoted the elasticity, chewiness, and adhesion of the emulsion, which was consistent with the rheological results. Compared with OPE, OGPE exhibited more excellent long-term storage stability, stronger gel strength, and higher viscosity, implying more remarkable emulsifying capability than that of OPE. In addition, OGPE also exhibited outstanding performance in regards to the protection and bioaccessibility of curcumin. The mechanism underlying such superior physicochemical properties and curcumin delivery capacity is related to the anchoring of the OTF—GA complexes at the oil–water interface, which was also confirmed by Cyro-SEM and CLSM. In conclusion, our work indicated that OTF—GA complexes produced a positive impact on the physicochemical properties and curcumin delivery capacity of oleogel-in-water Pickering emulsion. This would facilitate the precise designing of a Pickering emulsion with protein fibrils–polysaccharide complexes, providing new ideas for the large-scale application of a new oleogel-in-water Pickering emulsion.

## Figures and Tables

**Table 1:** Textural properties of OPE and OGPE.

Emulsion	Hardness (N)	Springiness (mm)	Cohesiveness (Ratio)	Gumminess (N)	Chewiness (mJ)	Adhesiveness (mJ)
OPE	1.21 ± 0.26 ^a^	0.30 ± 0.02 ^a^	0.66 ± 0.06 ^a^	0.87 ± 0.17 ^a^	0.26 ± 0.07 ^a^	0.20 ± 0.04 ^a^
OGPE	1.26 ± 0.16 ^a^	0.49 ± 0.03 ^b^	0.62 ± 0.03 ^a^	0.88 ± 0.11 ^a^	0.43 ± 0.07 ^b^	0.31 ± 0.01 ^b^

Note: Values are mean value ± standard deviation (*n* ≥ 3). Data in the same column with different superscript letters indicate significant differences (*p* < 0.05).

## Data Availability

The data presented herein are available on request from the corresponding author. The data are not publicly available due to privacy restrictions.

## Supplementary Materials

The following supporting information can be downloaded at: https://www.mdpi.com/article/10.3390/foods13091323/s1, Figure S1: Average droplet sizes of OPE and OGPE.
