# Supplementary material for: Ovotransferrin Fibril—Gum Arabic Complexes as Stabilizers for Oleogel-in-Water Pickering Emulsions: Formation Mechanism, Physicochemical Properties, and Curcumin Delivery
Source: Foods. 2024 Apr 26;13(9):1323. doi: 10.3390/foods13091323 (PMC11083342; doi:10.3390/foods13091323)
Supplement: Supplementary file 1 [file foods-13-01323-s001.zip › foods-2974897-supplementary.pdf]

foods

Supplementary Information

Ovotransferrin Fibril—Gum Arabic Complexes as Stabilizers for Oleogel-in-Water Pickering Emulsions: Formation Mechanism, Physicochemical Properties, and Curcumin Delivery

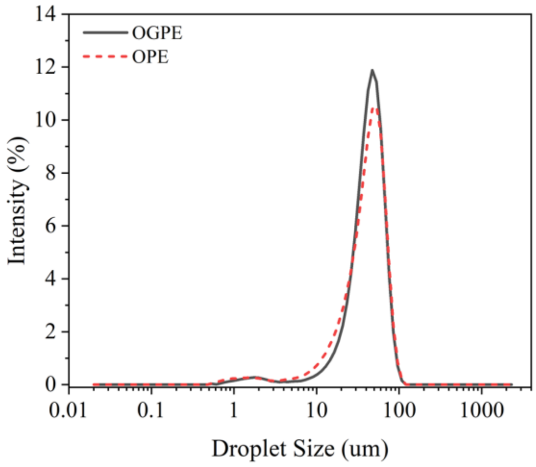

Figure S1. Average droplet sizes of OPE and OGPE.
